# Comparing an Unstructured Risk Stratification to Published Guidelines in Acute Coronary Syndromes

**DOI:** 10.5811/westjem.2015.6.16315

**Published:** 2015-10-20

**Authors:** Ann-Jean CC. Beck, Anouk Hagemeijer, Bess Tortolani, Bethany A. Byrd, Amisha Parekh, Paris Datillo, Robert Birkhahn

**Affiliations:** New York Methodist Hospital, Department of Emergency Medicine, Brooklyn, New York

## Abstract

**Introduction:**

Guidelines are designed to encompass the needs of the majority of patients with a particular condition. The American Heart Association (AHA) in conjunction with the American College of Cardiology (ACC) and the American College of Emergency Physicians (ACEP) developed risk stratification guidelines to aid physicians with accurate and efficient diagnosis and management of patients with acute coronary syndrome (ACS). While useful in a primary care setting, in the unique environment of an emergency department (ED), the feasibility of incorporating guidelines into clinical workflow remains in question. We aim to compare emergency physicians’ (EP) clinical risk stratification ability to AHA/ACC/ACEP guidelines for ACS, and assessed each for accuracy in predicting ACS.

**Methods:**

We conducted a prospective observational cohort study in an urban teaching hospital ED. All patients presenting to the ED with chest pain who were evaluated for ACS had two risk stratification scores assigned: one by the treating physician based on clinical evaluation and the other by the AHA/ACC/ACEP guideline aforementioned. The patient’s ACS risk stratification classified by the EP was compared to AHA/ACC/ACEP guidelines. Patients were contacted at 30 days following the index ED visit to determine all cause mortality, unscheduled hospital/ED revisits, and objective cardiac testing performed.

**Results:**

We enrolled 641 patients presenting for evaluation by 21 different EPs. There was a difference between the physician’s clinical assessment used in the ED, and the AHA/ACC/ACEP task force guidelines. EPs were more likely to assess patients as low risk (40%), while AHA/ACC/ACEP guidelines were more likely to classify patients as intermediate (45%) or high (45%) risk. Of the 119 (19%) patients deemed high risk by EP evaluation, 38 (32%) were diagnosed with ACS. AHA/ACC/ACEP guidelines classified only 57 (9%) patients low risk with 56 (98%) of those patients diagnosed with no ACS.

**Conclusion:**

In the ED, physicians are more efficient at correctly placing patients with underlying ACS into a high-risk category. A small percentage of patients were considered low risk when applying AHA/ACC/ACEP guidelines, which demonstrates how clinical insight is often required to make an efficient assessment of cardiac risk and established criteria may be overly conservative when applied to an acute care population.

## INTRODUCTION

Chest pain is the second most frequent complaint among patients presenting to the ED and is associated with the leading cause of death in the United States, coronary artery disease (CAD).^1^ It is estimated that about 117 million ED visits are made annually in the U.S., with just over 5% of those visits due to a primary complaint of chest pain.^2^ Approximately one third of the patients presenting with chest pain are diagnosed with acute coronary syndrome (ACS).^1^

ACS includes acute myocardial infarction (AMI) and unstable angina (UA). Acute MI is further differentiated by 12-lead-electrocardiogram (ECG) into two categories: non-ST-elevation myocardial infarction (NSTEMI) and ST-elevation myocardial infarction (STEMI). STEMI patients have ST elevations of ≥1mm in two or more consecutive leads on ECG findings. Patients with suspected UA or NSTEMI are identified by practice guidelines, clinical suspicion, patient risk factors, and cardiac enzyme determination.^4^,^5^ While patients with myocardial necrosis are identified by elevated cardiac enzymes, those ACS patients without evidence of myocardial necrosis remain difficult to identify because there is no true standard for the diagnosis of UA.^6^

The American Heart Association (AHA) in conjunction with the American College of Cardiology (ACC) and the American College of Emergency Physicians (ACEP) devised risk stratification guidelines to assist physicians with accurate and efficient diagnosis and management of patients with UA and NSTEMI.^7^ These guidelines suggest that a physician’s initial evaluation of a chest pain patient should verify the ‘likelihood that signs and symptoms represent an ACS secondary to CAD.’ This AHA/ACC/ACEP algorithm is considered the gold standard for risk stratifying a patient with suspected ACS secondary to CAD (Table 1). The guidelines established four categories to evaluate a potential cardiac patient: patient history, physical examination, ECG and cardiac markers. Each category contains specific criteria, which then determine a patient’s ACS risk as low, intermediate, or high.^7^–^9^

Theoretically, physicians can base their risk stratification on the AHA/ACC/ACEP guidelines. It is important to note that these guidelines simply provide a framework for the clinician to approach patients with suspected ACS. They are typically not used as a decision tool in clinical practice. Restraints on time, space, and resources in the ED compete with the need to efficiently and accurately diagnose a patient. We hypothesized that in the ED setting, providers are more likely to rely on their clinical experience when risk stratifying a patient for ACS. The objective of our study was to compare the point of care, unstructured ACS risk stratification value assigned by EPs to the score deduced from AHA/ACC/ACEP guidelines for ACS secondary to CAD. Our goal was to ascertain whether the practicing physician or the AHA/ACC/ACEP guidelines were more accurate in predicting a patient’s ultimate diagnosis of ACS.

## METHODS

### Study Design

This prospective observational cohort study analyzed the patient’s risk stratification for ACS as determined by an EP and by AHA/ACC/ACEP guidelines. All EPs evaluating patients with possible ACS calculated each patient’s risk for ACS. Using published AHA/ACC/ACEP guidelines, an independent observer determined a patient’s likelihood for ACS.

### Study Setting and Population

The setting for this study was the main ED of an urban, academic hospital with an annual census of approximately 90,000 ED visits per year. All patients ≥35 years of age presenting with a chief complaint of chest pain, and undergoing evaluation for ACS (indicated by cardiac-biomarker testing ordered) were prospectively enrolled. We defined exclusion criteria as patients who had a STEMI or new left bundle branch block (LBBB), left against medical advice (AMA), were sent to the ED by their primary physician or cardiologist for direct admission, or in cases where the EP played no role in patient disposition. Also excluded from enrollment were patients with unknown physician risk stratification or missing results from any cardiac testing. All patients were contacted at 48 hours and 30 days following the index ED visit to determine all cause mortality, unscheduled hospital/ED revisits, and objective cardiac testing performed.

Informed consent was not necessary because the scoring system has been incorporated as part of our electronic medical record and is our current standard of care. The information technology department integrated TIMI scoring and EP evaluation of patient ACS risk to auto populate ACS-related chief complaint notes (i.e. chest pain, and potential MI) as a mandatory field. A total of 641 patients were enrolled.

### Study Protocol

The treating EP evaluated all patients, and determined the diagnostic approach. EPs were required to document whether they suspected the patient to be at high, intermediate, or low likelihood for ACS, based on ECG findings, patient history, physical exam findings, and cardiac biomarkers. Two independent physicians reviewed patient charts using established AHA/ACC/ACEP guidelines and all available clinical data to identify the presence of UA, NSTEMI, as well as cardiac and non-cardiac death. These results are found in table 3.

### Outcome Measures

A standard database was used to record patient demographics, medical history, physical exam findings, cardiac biomarker values, objective cardiac testing, unstructured ACS risk stratification rating assigned by EP, likelihood of ACS by AHA/ACC/ACEP guidelines risk, final cardiology impression, 48-hour and 30-day follow-up information.

Our measures were the point-of-care ACS risk assessment by the EP, AHA/ACC/ACEP guidelines score, and the patient’s final diagnosis (scored as either ACS or no ACS).

### Data Analysis

We abstracted data for the study using double data entry for error checking. All charts were adjudicated by two EM resident physicians, using all available clinical data according to previously published AHA/ACC/ACEP guidelines to classify patients with regard to ACS diagnosis. In cases where the adjudication and diagnosis assigned by the treating physician were discordant, the medical records were reviewed by a panel comprised of a board-certified cardiologist and two board-certified EPs for consensus.

Electronic chart review included analysis of ED notes, index visit and hospital revisits, and cardiac test results including ECG, exercise stress test, pharmacologic stress test, myocardial perfusion and cardiac catheterization.

A diagnosis of ACS was noted if cardiac biomarkers were elevated due to myocardial injury (typical rise and fall of serial cardiac biomarkers), an ischemic defect was found by myocardial perfusion, a new or more narrowed stenosis of the coronary arteries was found upon catheterization per the cardiologist’s official report, revascularization was indicated, or if a diagnosis of ACS was documented in the patient’s discharge instructions.

Patient follow up at 48 hours included a phone call and review of inpatient charts. At 30 days the enrolled patient received up to three phone calls to connect with patient or caregiver. In addition, we reviewed all medical records through 30 days to identify any hospital revisits, significant cardiac events, and diagnostic cardiac testing.

## RESULTS

We identified 701 patients treated by 21 EPs who were eligible for the study. Sixty patients did not have an assessment of risk completed by the treating physician, leaving 641 patients in the study cohort. Overall, there was little concordance between the EP’s unstructured assessment used in clinical practice and the guidelines put forth by the AHA/ACC/ACEP task force. Physicians were more likely to assess a patient at low risk than the task force guidelines (40% vs 9%). While AHA/ACC/ACEP guidelines were more likely to classify patients as intermediate (45%) or high (45%) risk. Table 2 demonstrates the risk stratification of all 641 patients by AHA/ACC/ACEP guidelines and physician assessment. A comparison between the patient’s final ACS diagnosis and the relation to risk assessment value is provided in Table 3.

**Figure 1:**
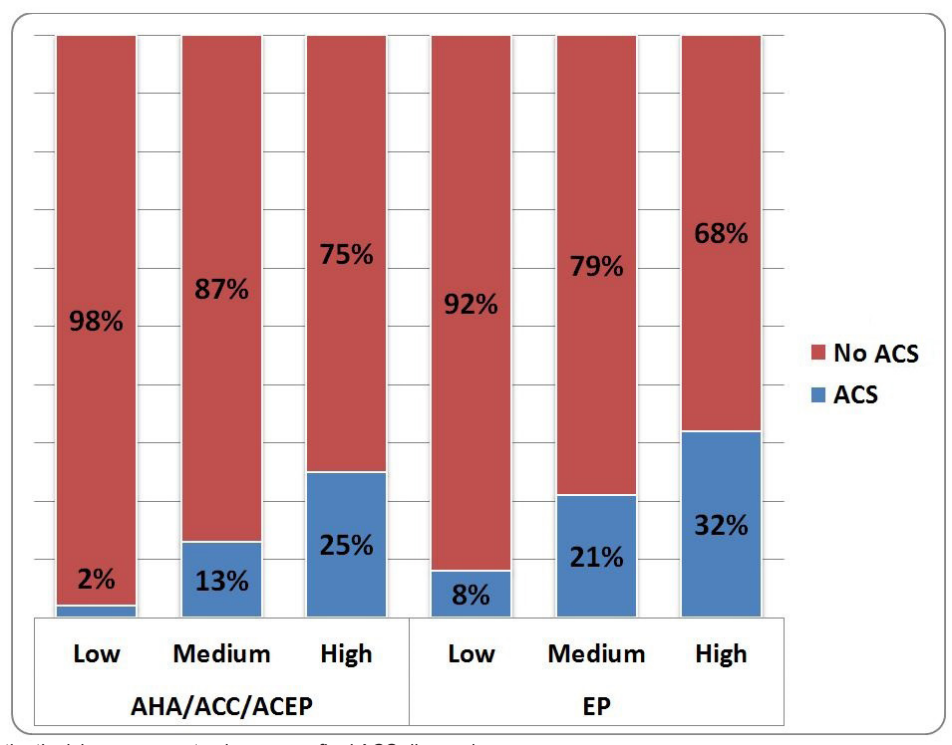
Patient’s risk assessment value versus final ACS diagnosis. *AHA*, American Heart Association; *ACC*, American College of Cardiology; *ACEP*, American College of Emergency Physicians; *ACS*, Acute Coronary Syndrome; *EP*, emergency physicians

**Figure 2:**
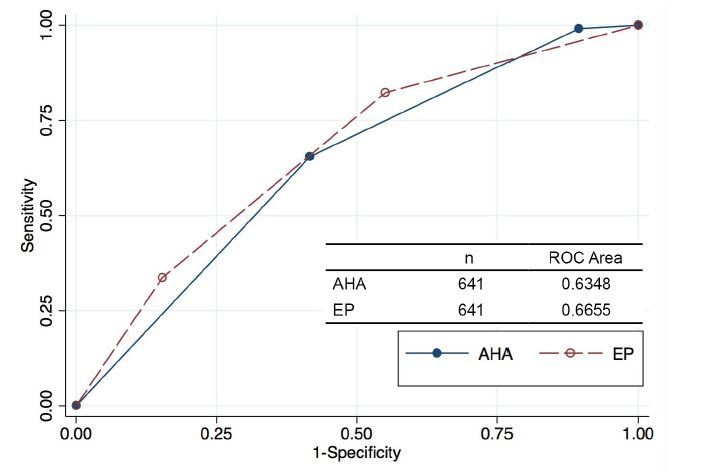
Receiver operating characteristic curve comparing AHA/ACC/ACEP to emergency physician risk stratification. *AHA*, American Heart Association; *ACC*, American College of Cardiology; *ACEP*, American College of Emergency Physicians; *EP*, emergency physicians; *ROC*, receiver operating characteristic

When considering the patient’s ACS diagnosis and its relation to the risk assessment value (Table 3), AHA/ACC/ACEP guidelines proved better at identifying low-risk patients who did not have ACS (only 2% had ACS vs. 8% for EPs), while EPs proved better predictors of high-risk patients who in fact had ACS (68% had no ACS vs 87% for AHA/ACC/ACEP guidelines). Of all enrolled patients, 119 (17%) were determined by the EP to be at high risk for ACS; 38 (32%) of the 119 high-risk patients were diagnosed with ACS. The AHA/ACC/ACEP guidelines classified 294 (45%) patients high risk, with 74 (25%) of those patients diagnosed with ACS. AHA/ACC/ACEP guidelines classified only 57 (9%) patients low risk, with 56 (98%) of those patients diagnosed with no ACS. In contrast, physicians classified 257 (40%) of the sample as low risk for ACS, of whom 20 (8%) actually had ACS. Chi-square test of independence identified a difference in physician and AHA/ACC/ACEP scores, and their relation to ACS diagnosis (p≤0.05). Graphical representation of the physician risk assessment and guideline classification stratified by final diagnosis is shown in Figure 1. The receiver operating characteristic curves showing the performance for either the EP clinical impression or the AHA/ACC/ACEP scores for identifying patients with underlying ACS are shown in Figure 2.

Within 48 hours, 67% of patients discharged to home received follow-up phone calls. At 30 days, follow up on 86% of patients was obtained by phone, EMR check for return visits, contacting PMD (if known), or mail.

## DISCUSSION

The prevalence of patients presenting to the ED with chest pain of cardiac origin results in many non-cardiovascular specialists evaluating and managing this patient population. Although the majority of patients presenting to the ED with chest pain do not have a life-threatening condition, the EP needs to efficiently and accurately differentiate between those patients requiring urgent treatment and those who will not warrant hospital admission.^13^ AHA/ACC/ACEP guidelines may prove worthwhile for use in a primary care setting, but our study reveals these guidelines may not be as valuable a tool for use in the ED.

Our study shows that in the ED, physicians are more adept at correctly placing patients with underlying ACS into a high-risk category. AHA/ACC/ACEP guidelines place a greater number of patients into the high-risk category than physicians (294 (45%) vs 119 (19%)), but fewer of these patients have underlying ACS (25% vs 32%). Furthermore, only a small percentage of patients (57/641 (9%)) were assessed as low risk by task force guidelines. The task force guidelines’ predilection to assess patients as low risk may be useful in primary care where the clinical decision is whether a patient should undergo further cardiac testing. However, in the ED the decision point is not whether a patient should undergo cardiac testing, but if that testing should be done as an inpatient or outpatient. In the primary care setting, it is more useful to have a broader net since the consequence of a missed diagnosis of ACS would be an undiagnosed cardiac condition. In our cohort, all patients had serial cardiac biomarkers to assess for an acute ischemic event (unstable angina would still be ACS with negative biomarkers). Misclassification of a patient with underlying ACS into a low-risk category would be the difference between inpatient and outpatient cardiac testing. In either case, cardiac testing is recommended at time of disposition. In contrast, the use of AHA/ACC/ACEP guidelines to guide clinical decision-making would have quadrupled the use of inpatient hospital resources at our institution. A plausible explanation for this observed trend is that AHA/ACC/ACEP guidelines require only one criterion to be met for a patient to be grouped into a higher risk assessment category, whereas the physician considers multiple factors when assessing a patient for ACS. AHA/ACC/ACEP guidelines were developed for a national population that is approximately 72% Caucasian; in the urban teaching hospital where this study was conducted, our study population was far more racially diverse with 40% Black, 30% White, 24% Hispanic, and 6% Asian. This may have contributed to a difference in application of guidelines in the study population.

The impact of clinical insight when assessing cardiac risk is demonstrated by the comparison between EP and AHA/ACC/ACEP guidelines and ACS final diagnosis. The results show that EPs correctly assigned 7% more patients with ACS to the appropriate high-risk category than the AHA/ACC/ACEP guidelines. The AHA/ACC/ACEP guideline correctly assigned 6% more patients without ACS to the appropriate low-risk category (Table 3).

As ED crowding continues to be an obstacle for hospitals and EPs, it is crucial to develop a better method to evaluate chest pain patients. If the AHA/ACC/ACEP guidelines are the criterion reference for risk stratifying a patient with chest pain in the ED, the possibility of further hampering patient flow needs to be considered.

## LIMITATIONS

Our study has a few notable limitations. First, the study took place at one clinical center. This limits the ability to generalize findings to other clinical centers as they may have different staffing, patient demographics, and technology/instruments available for use. As an urban teaching hospital, our study may show different trends than rural or non-teaching facilities.

We made attempts to conduct 48-hour and 30-day follow up of all patients by chart review, and three attempts were made by phone. Through these methods of follow up we were not able to account for patients who sought care at another hospital, provided an incorrect phone number, or whom we were unable to reach.

A final limitation to our study was the lack of verification that the diagnosis of ACS was a primary event. This information could skew data, as patients who have had more than one event are likely to present differently than someone experiencing chest pain for the first time, and this presentation would likely influence a physician’s ACS risk assessment.

## CONCLUSION

In the ED, more so than anywhere else in medicine, the need to efficiently and accurately diagnose a patient comes into direct conflict with limitations on time, space, and resources. Our study suggests that physicians were more efficient at placing patients with underlying ACS correctly into a high-risk category. At the lower end of the scale, clinicians using an unstructured risk assessment translated this efficiency into a much broader group classified as low risk than would have been recommended by existing AHA/ACC/ACEP guidelines. Although the guidelines would have classified just one patient with underlying ACS as low risk in this cohort, it would have done so at the cost of a four-fold increase in the number of patients requiring more ED and hospital resources. The guidelines meant to inform clinicians when evaluating patients with suspected ACS may be overly conservative when applied to the ED in an era of crowding.

## Figures and Tables

**Table 1:** AHA/ACC/ACEP risk stratification for ACS.

	High likelihood	Intermediate likelihood	Low likelihood
	
Feature	Any of the following	Absence of high-likelihood features and presence of any of the following:	Absence of high- or intermediate-likelihood features but may have:
History	Chest or left arm pain or discomfort as chief symptom reproducing prior documented angina Known history of CAD, including MI	Chest or left arm pain or discomfort as chief symptom Age >70 years Male sex Diabetes mellitus	Probable ischemic symptoms in absence of any of the intermediate likelihood characteristics Recent cocaine use
Examination	Transient MR murmur, hypotension, diaphoresis, pulmonary edema, or rales	Extracardiac vascular disease	Chest discomfort reprduced by palpation
ECG	New, or presumably new, transient ST-segment deviation (≥0.1 mV) or T-wave inversion in multiple precordial leads	Fixed Q waves ST depression 0.05 to 0.1mV or T-wave inversion >0.1mV	T-wave flattening or inversion <0.1mV in leads with dominant R waves or normal ECG
Cardiac markers	Elevated cardiac Tnl, TnT, or CK-MB	Normal	Normal

*AHA,* American Heart Association, *ACC*, American College of Cardiology; *ACEP,* American College of Emergency Physicians; *ACS,* acute coronary syndrome; *CAD*, coronary artery disease; *ECG,* electrocardiogram; *CK-MB*, MB fraction of creatine kinase; *MI*, myocardial infarction; *MR*, mitral regurgitation; *Tnl*, troponin; *TnT*, troponin T

Reproduced from Anderson et al.^12^

**Table 2:** AHA/ACC/ACEP guidelines versus emergency physician (EP) risk stratification for ACS.

Total N=641	Low	Intermediate	High
AHA/ACC/ACEP	57 (9%)	290 (45%)	294 (45%)
EP	257 (40%)	265 (41%)	119 (19%)

*AHA*, American Heart Association; *ACC*, American College of Cardiology; *ACEP*, American College of Emergency Physicians; *ACS*, Acute Coronary Syndrome

**Table 3:** Comparison of ACS positive diagnosis by EP and AHA/ACC/ACEP guidelines.

	EP			
	
	Low	Medium	High	Total
AHA/ACC/ACEP				
Low	0	1	0	1/57
Medium	13	23	2	38/290
High	7	31	36	74/294
Total	20/257	55/265	38/119	-

*AHA*, American Heart Association; *ACC*, American College of Cardiology; *ACEP*, American College of Emergency Physicians; *ACS*, acute coronary syndrome; *EP*, emergency physicians
